# Spatial Transcriptomics: A Powerful Tool in Disease Understanding and Drug Discovery

**DOI:** 10.7150/thno.95908

**Published:** 2024-05-11

**Authors:** Junxian Cao, Caifeng Li, Zhao Cui, Shiwen Deng, Tong Lei, Wei Liu, Hongjun Yang, Peng Chen

**Affiliations:** 1 Beijing Key Laboratory of Traditional Chinese Medicine Basic Research on Prevention and Treatment for Major Diseases, Experimental Research Center, China Academy of Chinese Medical Sciences, Beijing 100700, China.; 2 Institute of Chinese Materia Medica, China Academy of Chinese Medical Sciences, Beijing 100700, China; 3 Institute of Basic Theory for Chinese Medicine, China Academy of Chinese Medical Sciences, Beijing 100700, China.; 4 Analysis of Complex Effects of Proprietary Chinese Medicine, Hunan Provincial Key Laboratory, Yongzhou City, Hunan Province, China.

## Abstract

Recent advancements in modern science have provided robust tools for drug discovery. The rapid development of transcriptome sequencing technologies has given rise to single-cell transcriptomics and single-nucleus transcriptomics, increasing the accuracy of sequencing and accelerating the drug discovery process. With the evolution of single-cell transcriptomics, spatial transcriptomics (ST) technology has emerged as a derivative approach. Spatial transcriptomics has emerged as a hot topic in the field of omics research in recent years; it not only provides information on gene expression levels but also offers spatial information on gene expression. This technology has shown tremendous potential in research on disease understanding and drug discovery. In this article, we introduce the analytical strategies of spatial transcriptomics and review its applications in novel target discovery and drug mechanism unravelling. Moreover, we discuss the current challenges and issues in this research field that need to be addressed. In conclusion, spatial transcriptomics offers a new perspective for drug discovery.

## 1. Introduction

Spatial transcriptomics (ST) is used to explore the spatial gene expression patterns of cells or tissues. It combines traditional histological techniques with high-throughput RNA sequencing to visualize and quantitatively analyse the transcriptome with spatial distribution in tissue sections 1. In single-cell sequencing, cells are prone to dissociate in suspension, leading to the loss of positional information 2. However, ST preserve the spatial information of RNA in tissue sections by mapping it to specific spatial locations and subsequently subjecting it to high-throughput sequencing, thus providing spatial information on the transcriptome 3. ST can help uncover cellular and tissue heterogeneity, enabling a deeper understanding of the diversity within tissues, identification of signature genes for specific cell types, and exploration of intercellular interactions 4. This technology offers a novel perspective for understanding the spatial variations and distributions of gene expression.

With the emergence of spatial transcriptomics, our ability to understand drug actions and discover new medications has been revolutionized. By integrating ST, gene expression profiles and spatial distribution data can be obtained while preserving the integrity of tissue structures 5. This study allows for a deeper understanding of the processes and molecular mechanisms of drug action within the body, further advancing research into the dynamic panorama of drug effects. The application of ST in the field of drug research has significantly broadened our perspectives, paving the way for groundbreaking drug discoveries with far-reaching impacts.

**Figure 1:**
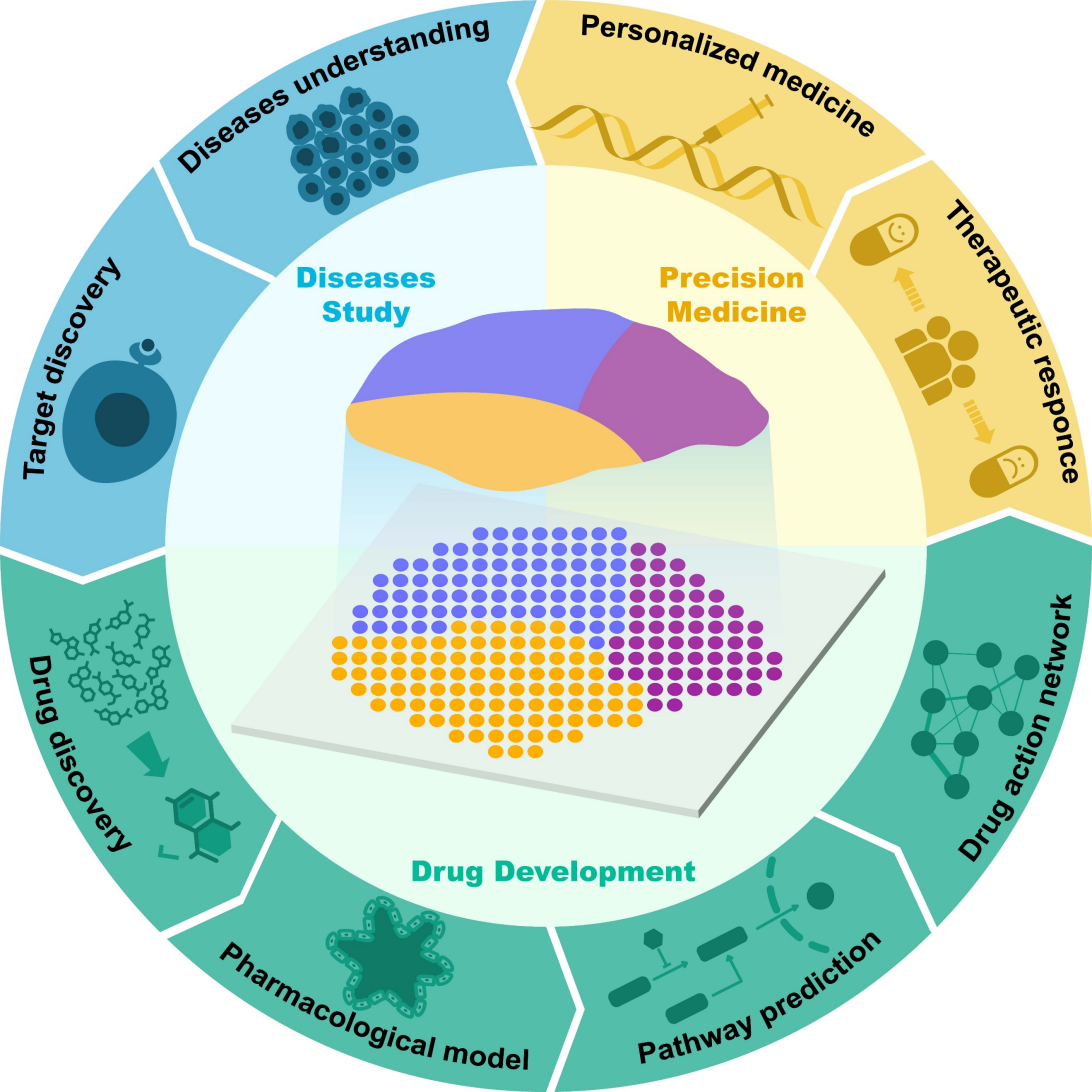
Application of spatial transcriptomics in drug research and development.

Currently, the applications of single-cell transcriptomics, proteomics, metabolomics, and genomics in drug discovery have been extensively reviewed 6-9. However, most of the current reviews are still focused on discussing the application of ST in disease studies, and there is still a lack of comprehensive summaries concerning the application of ST in drug discovery and development. Therefore, in this article, we review the latest advancements in the use of ST in drug discovery and development. First, we outline the key technological and analytical research progress of current ST, encompassing methods based on microanatomy, in situ capture, and other approaches, while elucidating the strengths and limitations of each method. Subsequently, we introduce the applications of ST in disease research and target discovery and discuss the advancements and limitations of current ST analysis technologies. Additionally, we review classic cases of ST applications in drug development research, discussing their crucial contributions to new drug discovery, pharmacological model construction, drug action pathway research, and spatially dynamic drug action studies. We also discuss the application of ST in personalized therapy (Figure 1). Finally, we provide prospects for the future opportunities and challenges faced by ST in drug development.

## 2. Strategies for spatial transcriptomics

**Figure 2:**
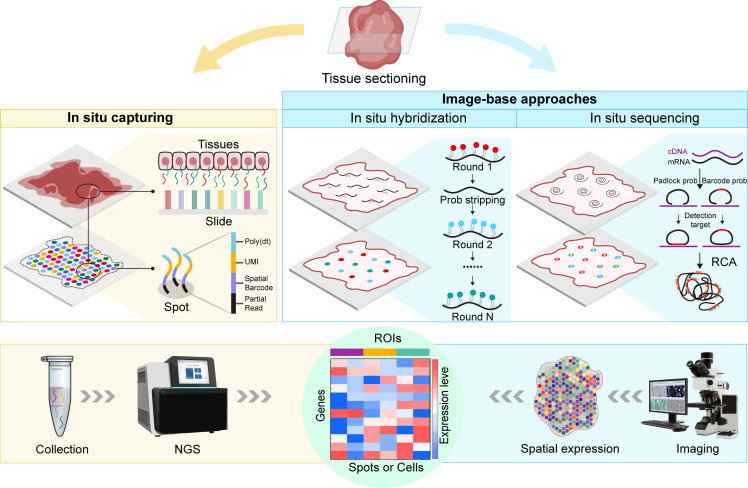
**Current mainstream spatial transcriptomics methods.** ISC involves the specific binding of mRNA from corresponding cellular locations with spots or beads on slides carrying capture probes, followed by further NGS of the captured RNA molecules. ISC offers high sensitivity and specificity, enabling the detection of rare transcripts and precise spatial localization, while achieving single-cell resolution with this technology is currently challenging. Image-based methods, including ISH and ISS. ISH involves the specific hybridization of RNA in tissues with multiple fluorescently labelled short oligonucleotide probes, reflecting the abundance and spatial localization of specific gene transcripts. ISH detects low-abundance transcripts with high sensitivity and relatively high resolution, while the limited signal-to-noise ratio during imaging necessitates high-magnification imaging systems, limiting the area of detection. ISS involves the hybridization of padlock probes with cDNA, followed by RCA to form RCP. ISS allows for the sequencing of RNA molecules directly on tissue sections, providing higher-resolution data, while it has lower sensitivity, and padlock probes may introduce significant probe-specific biases, increasing the complexity of image processing.

As omics technologies flourish, single-cell transcriptomics has greatly advanced drug discovery and development. However, single-cell transcriptomics relies on the isolation of individual cells, inevitably leading to the loss of the original spatial information within the studied samples. The emergence of ST overcomes this limitation, allowing scientists to obtain spatial distribution information on the transcriptome within research samples. The essence of ST lies in unravelling the spatial localization of detected RNA molecules. Since the concept of ST was first proposed by Joakim Lundeberg's team in 2016 1, ST has integrated various technologies and developed diverse technical approaches, with both throughput and resolution rapidly increasing. Currently, ST has extensive applications in various fields, such as cancer research 10, developmental biology 11, pathology 12, and toxicology 13. Notably, in 2021, ST was recognized as the "Method of the Year" by Nature Methods 14. In this section, we provide a comprehensive review of mainstream ST research strategies and summarize the strengths and limitations of these methods (Figure 2 and Table 1).

### 2.1 In situ capture (ISC)

Fundamentally, in situ capture (ISC) involves the in situ labelling of RNA molecules within designated regions of interest (ROIs) using spatial barcodes before library preparation, followed by the extraction and spatially resolved sequencing of the captured RNA molecules via next-generation sequencing (NGS). The ST strategy was initially proposed by Joakim Lundeberg's team, who applied this method to reveal spatial information on gene expression in mouse brain and human breast cancer tissues 1. In this approach, the authors immobilize reverse-transcription oligo primers with spatial barcodes onto a chip and subsequently perform tissue sectioning and reverse transcription to obtain cDNA. The spatial positions were recorded by comparing the fluorescence signal of fluorescently labelled DNA with hematoxylin and eosin staining of the tissues. Subsequently, next-generation sequencing was performed ex situ, and the spatial barcode-containing RNA-seq data were aligned to the corresponding positions in the image to visualize the transcriptome information. In 2018, 10X Genomics acquired this technology and launched a commercial ST platform called "Visium Spatial Gene Expression", which improved the resolution from the original 100 µm spot diameter to 55 µm, reduced the spot-to-spot distance from 200 µm to 100 µm, and shortened the processing time 15.

In the Slide-seq method 16, microbeads covered with DNA barcodes are densely arrayed onto a glass slide, with each microbead bearing a distinct spatial identifier 17. Subsequently, RNA from tissue sections can be captured on these microbeads, followed by sequencing of the barcode RNA library using single-cell RNA sequencing (scRNA-seq). The scRNA-seq results are then mapped onto the Slide-seq data, allowing for comprehensive integration of spatial and single-cell transcriptomics information with non-negative matrix factorization regression (NMFreg) 18. The size of these microbeads is only 10 μm, allowing minimal lateral diffusion of mRNA from the tissue to the microbeads. This innovation enables the visualization of high-resolution, high-throughput ST. In 2020, the research team introduced a new generation of Slide-seq, named Slide-seqV2. Compared with its predecessor, Slide-seqV2 has undergone enhancements in library preparation, microbead synthesis, and array indexing. These improvements have resulted in a tenfold increase in RNA capture efficiency for Slide-seqV2, offering a substantial advancement over the first generation 19.

Shortly after the publication of Slide-seq, another technique utilizing even smaller barcode-bearing microbeads emerged, named high-definition spatial transcriptomics (HDST) 20. HDST employs a similar principle as Slide-seq but features high-density microbeads with a diameter as small as 2 μm on the chip array. Traditional ST platforms mainly face the problem of insufficient spatial resolution, as multiple cells are captured in one spot. The latest ST platforms provide smaller and denser spots, which theoretically have the potential to capture more mRNA in an area, yielding an even higher spatial resolution 21. However, higher spatial resolution does not necessarily improve the capacity to resolve individual cells. Two different cells can still be captured even if subcellular resolution (less than 10 μm) is achieved; therefore, the main challenge of current high spatial resolution platforms lies in cell separation and spot allocation issues 22.

In contrast to strategies involving the embedding of spatial information with microbeads, APEX-seq is an ST method that relies on the direct labelling of RNA with the peroxidase enzyme APEX2 23. APEX-seq enables the precise localization of endogenous RNA within individual live cells, achieving the remarkable resolution of single-nucleotide RNA sequence information 24. Nevertheless, a drawback of APEX-seq lies in the requirement for APEX to be recombinantly expressed within target cells, thereby limiting its applicability within normal human tissues.

The Seq-Scope is a submicron resolution ST based on the Illumina sequencing platform that involves a two-round sequencing process 25. In the first round, cDNA is solid-phase amplified to generate spatial barcodes on the capture array 26. In the second round, mRNA molecules captured on the physical array are released and sequenced. This approach achieves a spatial resolution of 0.5 to 0.8 μm.

The progression of other in situ capture-based ST methods remains ongoing. Noteworthy examples include DBIT-SEQ 27, which leverages microfluidic barcode labelling for spatial transcriptomic sequencing. Pixel-seq achieves a high-density DNA chip with a resolution of 1 μm at lower cost by repetitive printing on the surface of a polyacrylamide gel 28. Stereo-seq uses a randomly synthesized 25-nucleotide sequence as a spatial coordinate (coordinate identity, CID), which is amplified through rolling-circle replication to form DNA 29. These DNBs are then attached to the Stereo-seq Chip, followed by sequencing of the DNBs on the chip using the DNBSEQ sequencer 30. The Stereo-seq chip used for capturing mRNA has regularly arranged spots, with each spot having a diameter of approximately 220 nm and a center-to-center distance between spots of 500 nm. The GeoMx Digital Spatial Profiler (DSP) introduced by NanoString Technologies is a probe-based technology that attaches DNA oligos to antibodies or RNA, with each DNA oligo corresponding to a target 31. It can detect up to 96 protein targets or more than 1000 RNA targets. DSP enables users to select ROIs based on tissue outlines on a single slide while maintaining spatial context with cellular-level resolution. The ongoing development of these methodologies holds promise for advancing our understanding of spatial gene expression patterns.

### 2.2 Imaging-based approaches

Imaging-based ST can be taxonomically classified into two primary categories based on the methodology employed for RNA detection: in situ sequencing (ISS) and in situ hybridization (ISH). The fundamental principle of ISH involves the utilization of complementary fluorescently labelled probes to perform in situ hybridization with target RNA, enabling both quantitative detection and spatial localization of the target RNA molecules 32. The origins of ISH can be traced back to 1969 when Pardue and Gall pioneered the use of isotopically labelled nucleic acid probes to ascertain the abundance and spatial distribution of DNA and RNA within tissues and cells, a pioneering technique also known as fluorescence in situ hybridization (FISH) 33. Early FISH approaches could only detect individual transcripts. Femino et al. combined FISH with digital microscopy imaging, targeting RNA molecules with oligonucleotide probes synthesized with five fluorescent dyes per molecule, thus achieving the detection of single RNA molecules 34. While this method exhibits high sensitivity and achieves subcellular resolution, it is limited by its resolution, allowing the simultaneous measurement of only a few mRNA molecules. Subsequently, Raj et al. improved this method by using multiple short, single-labelled oligonucleotide probes to detect each mRNA type, achieving more efficient in situ hybridization, and they named this method single-molecule fluorescence in situ hybridization (smFISH) 35. While smFISH offers high sensitivity and subcellular spatial resolution, it is limited in its capacity to target only a few genes simultaneously due to spectral overlap constraints in standard microscopy. Therefore, researchers have turned their attention to multiplexed smFISH techniques, such as sequential hybridization FISH (seqFISH) introduced in 2018, which involves multiple rounds of hybridization, imaging, and probe stripping, allowing for the sequential detection of individual transcripts 36. However, repeated hybridization of multiple transcripts is expensive and time-consuming. Cyclic-Oroboros smFISH (osmFISH) is a cyclic hybridization method involving multiple rounds of hybridization with probe removal after each imaging round before the next hybridization 37. The multiplexed error-robust fluorescence in situ hybridization (MERFISH) technique, introduced in 2015, uses a combination of FISH labelling and error-robust encoding, enabling the analysis of hundreds or even thousands of RNA molecules within cells 38. Combining MERFISH with super-resolution microscopy significantly increases the total density of measurable RNA 39. DNA microscopy is a novel imaging method introduced in 2019 that relies on molecular thermodynamic entropy imaging instead of optical devices for spatial capture through the encoding of physical images into DNA and the generation of physical images of the original transcripts with precise sequence information, enabling the observation of cells at the genomic level 40. Recently developed electrically enhanced fluorescence in situ hybridization (EEL FISH) methods can be used to capture RNA on a glass surface via electrophoresis and enable imaging after tissue removal, thereby facilitating accelerated imaging while preserving single-cell resolution 41.

Current in situ sequencing (ISS) technology usually relies on RNA as a probe ligation template. ISS employs padlock probes that hybridize to target RNA in tissues, circularize after hybridization, and produce numerous copies of the original probe through rolling circle amplification (RCA), which are then sequenced and visualized using fluorescence or chromogenic detection methods 42,43. With the aid of microfluidic platforms, ISS technology has achieved automation, significantly enhancing efficiency and enabling the simultaneous detection of thousands of RNA species 44. However, ISS technology based on gap-fill padlock probes is limited to targeted sequencing. Fluorescent in situ RNA sequencing (FISSEQ) achieves non-targeted detection of more than 8,000 RNA species in fibroblasts by reading 27 bases of each transcript 45. Spatially resolved transcript amplicon readout mapping (STARmap) combines ISS technology with hydrogel chemistry, utilizing dynamic annealing and cross-linking to reduce sequencing errors, enabling the simultaneous analysis of more than one thousand genes and providing three-dimensional images of complete tissues 46. While sequencing technologies based on padlock probes have low efficiency in reading barcodes, BaristaSeq combines the Illumina sequencing platform with a simultaneous synthesis and sequencing strategy to enhance sequencing efficiency and accuracy 47. Subsequently, researchers further developed BARseq based on this approach 48. Moreover, many companies have also introduced commercial platforms based on image-based ST and applied them to research, including MERSCOPE 49, Xenxium 50, GeoMX, and CosMX 51.

### 2.3 Other approaches

In addition to the mainstream methods mentioned before, as an emerging research field, new research strategies in the field of ST continue to emerge. For example, microscopic isolation approaches involve cutting tissue regions under a microscope, isolating individual cells or entire target areas, and then combining them with NGS for ST 52. Laser capture microdissection (LCM) uses a laser beam to cut tissue regions under a microscope for spatial transcriptomic analysis 52. Geographical position sequencing (GEO-seq) technology integrates LCM with scRNA-seq to analyse the entire transcriptome of a small number of cells while preserving their original spatial positions through zip-code genes 53. Tomo-seq involves freezing tissue for sectioning and performing ex vivo transcriptional amplification on each section, thereby obtaining three-dimensional spatial transcriptomic information 54. Topographic single-cell sequencing (TSCS) technology involves placing tissue slices on glass slides, using an LCM system to capture individual cells from the tissue slices while concurrently recording their coordinates, and subsequently performing next-generation sequencing (NGS) and single-nucleus sequencing 55. A spatial transcriptomic approach based on transcriptome in vivo analysis (TIVA) uses light-activated biotin tags to enter living cells through cell-penetrating peptides, photoactivates the target regions to capture mRNA, and then performs RNA-seq 56,57. Only a few cells can be analysed at a time via this method, which limits its application in high-throughput ST. Additionally, researchers have developed an ST method named RAINBOW-seq, which is specifically designed for colony analysis 58. While these methods may currently have various limitations, the field of ST is experiencing rapid growth, and over time, these technologies may prove to be unexpectedly valuable in specific research domains.

## 3. ST for disease understanding and novel target identification

**Figure 3:**
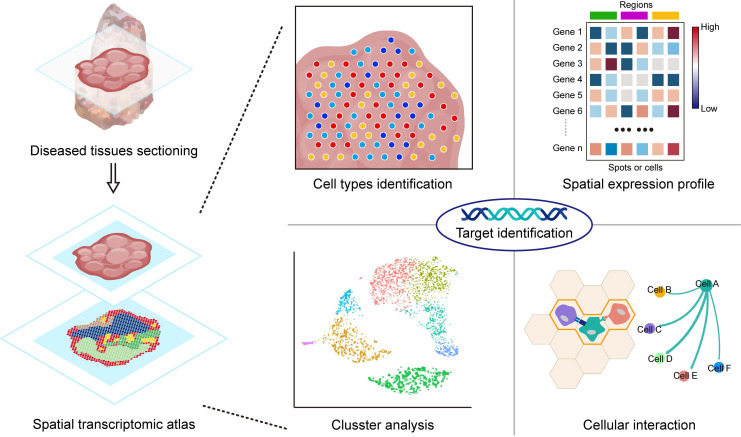
** Application of spatial transcriptomics in target identification.** By identifying cell types through spatial ST, it is possible to reveal the distribution of specific cell types within tissues and understand their function and state. The spatial expression profile depicts the expression of mRNAs in spatial dimensions, aiding in the identification of characteristic DEGs involved in disease processes. Clustering analysis helps in discovering specific cell populations associated with diseases that may be closely linked to the occurrence, progression, or prognosis of the disease. By analysing interactions between cells, key interactions involved in cell communication and signal transduction can be identified. Integrating this information can provide insights into the process of disease occurrence and reveal potential therapeutic targets.

As multi-omics technologies continue to progress, the landscape of drug target discovery has evolved significantly 59. Multi-omics approaches, such as genomics 60, proteomics 61, and transcriptomics 62, offer an in-depth perspective for unravelling the molecular mechanisms underlying diseases. In contrast to conventional transcriptomics, the emerging field of ST offers distinct advantages by preserving spatial information within transcripts 63. Mapping the gene expression patterns in specific tissues and cell regions enables the identification of interactions between different cell types in different areas, including signalling pathways, cell adhesion, and cell-cell communication 64,65 (Figure 3). This innovative approach revealed genes exhibiting heightened expression levels in specific cells or subcellular structures within regions of interest, revealing potential targets for therapeutic intervention 66,67. In the ensuing section, our focus centers on an extensive review of the applications of ST in the discovery of novel drug targets.

### 3.1 Cancers

Tumor heterogeneity results in the high complexity and diversity of malignant tumors during their evolution, while drug resistance caused by tumor heterogeneity is a major challenge in current cancer research and antitumour drug discovery 68-70. ST demonstrated excellent performance in identifying cell types within different tumor regions. Glioblastoma (GBM) is a common primary malignant tumor. However, current therapeutic modalities have shown limited efficacy in improving patient survival rates. ST analysis revealed the overexpression of EphA3 and ephrinA5 in certain regions of GBM tumors, with mutually exclusive expression patterns in different tumor regions, which are associated with the formation and induced differentiation of GBM cells 71. EphrinA5 is a high-affinity ligand of EphA3, and EphA3 was identified as a target in GBM in a previous study 72. Therefore, targeting the regulation of EphA3 and ephrinA5 could offer a more sustained and effective therapeutic approach. The integration of ST with single-cell transcriptomics has enabled the discovery and localization of novel cellular subtypes within tumors. Through scRNA-seq, a specific tumor-specific keratinocyte (TSK) has been identified as a distinct cell type unique to cutaneous squamous cell carcinoma, and the frontier heterogeneity of this cell cluster has been elucidated with ST, revealing its pivotal role as a hub for intercellular communication 73.

ST offers a significant advantage by facilitating comprehension of the spatial arrangement of diverse cell types within the tumor microenvironment (TME) and furnishing crucial spatial details concerning the transcriptomic landscape of tumor cells. This capability substantially enhances our understanding of cellular interactions and spatial impacts within the TME. Metastasis is a crucial factor contributing to high cancer mortality rates and is closely associated with drug resistance 74. Therefore, identifying key genes and pathways involved in metastasis may reveal potential therapeutic targets for cancer treatment. Brain metastasis (BrMs) is the main component of malignant cancers of the central nervous system. To understand the molecular and cellular mechanisms of lung cancer brain metastasis, researchers have mapped a comprehensive transcriptional atlas of brain metastasis in non-small cell lung cancer with ST analysis on tumor samples from 44 patients 75. Analysis of the ROIs revealed that the TME plays a pivotal role in metastasis and that extensive remodelling occurs during the metastatic process. Within the TME, there is a notable increase in fibrotic factors, fostering an immune-suppressed and fibrotic milieu that supports the development of BrMs. Elevated levels of fibrotic factors such as PDGFRβ, CXCR4, and TGFB1 are associated with promoting tumor growth and angiogenesis 76. Consequently, the adjustment of treatment strategies based on the fibrotic state has become crucial, but targeting fibrosis-regulating factors in fibrotic BrMs has emerged as a potential therapeutic approach. Pancreatic ductal adenocarcinoma is a common digestive tract cancer known for its rapid metastasis and poor prognosis. Through whole-transcriptome digital spatial profiling, a neural-like progenitor molecular program present in cancer cells that persists after treatment has been identified. These unique receptor-ligand interactions confer resistance to therapies on these tumor cells 77. This study revealed that this gene is a promising potential target for novel therapies.

### 3.2 Neurological diseases

Neurological disorders encompass a variety of cell types, and previous research utilizing scRNA-seq has offered novel insights into disease comprehension at the molecular level. As most complex neurological diseases involve interactions of different brain regions, ST can significantly advance target discovery by discerning transcriptional information and identifying cell types in distinct functional regions. For example, ST has been applied to elucidate the underlying mechanisms and identify potential therapeutic targets for conditions such as amyotrophic lateral sclerosis (ALS) 78, Alzheimer's disease (AD) 79, multiple sclerosis (MS) 80, and Parkinson's disease (PD) 81. ST has been applied to construct a spatiotemporal map of ALS, wherein a discernible reduction in the expression module associated with various biological pathways, encompassing myelination, retrograde endocannabinoid signalling, and WNT signalling, was observed in spinal cord slices located proximal to the onset of symptoms 78. This observation suggests the potential utility of myelination signalling modulators as a therapeutic avenue for ALS. Earlier research leveraged the ST to uncover novel targets within the hippocampal and olfactory bulb layers of AD mouse models 82. Furthermore, subsequent studies employing ST identified differentially expressed genes in layer 2/3 of the middle temporal gyrus among postmortem individuals with and without AD 83. Additionally, the use of the ST has provided insights into the treatment of progressive MS in conjunction with proteomic analysis. Through the tracking of changes in gene and protein pairs across spatially distributed stages of neurodegeneration, novel target candidates for progressive MS, including GPR37L1, TYRO3, SIRPA, and FGFR3, were identified 80. Alterations in gene expression within distinct regions of the PD-affected hippocampus have also been revealed in the ST. Differential expression gene analyses based on ST imply that upregulated genes primarily contribute to processes such as neuron-to-neuron synapse function, vesicle-mediated transport in synapses, the calcium signalling pathway, and pathways associated with neurodegenerative diseases. Conversely, downregulated genes are predominantly linked to ATP metabolism and the GnRH signalling pathway 81. The key genes identified in this study hold promise as potential novel therapeutic targets for PD treatment. In addition to its applications in the study of neurodegenerative diseases, ST analysis of cortical layers in a depression-like macaque model revealed discrete gene expression regions involved in the regulation of diverse emotional states, suggesting potential targets for the modulation of depressive emotions 84. Furthermore, the ST has shed light on the complexity of schizophrenia-associated genes, revealing intricate intracellular signalling networks rather than isolated individual genes 85. Thus, the exploration of multitarget therapeutic approaches for schizophrenia merits serious consideration. Moreover, the CCL5/CCR5 signalling pathway is a specific pathway in the hippocampal region of epileptic mice, and blocking CCL5/CCR5 signal transmission can alleviate hippocampal lesions, indicating that the CCL5/CCR5 signalling pathway is a potential therapeutic target for epilepsy 86.

### 3.3 Cardiovascular diseases

Cardiovascular disorders primarily comprise pathologies that impact the cardiac and vascular systems. In the field of cardiac diseases, ST has been harnessed for the purpose of identifying potential therapeutic targets for viral myocarditis 87, myocardial infarction (MI) 88, heart failure 89, and arrhythmogenic cardiomyopathy (ACM) 90. ST enables the creation of detailed cardiac maps across various anatomical regions, advancing our understanding of cardiac development and disease at the molecular level 91. For example, in the early stages after MI, mechanosensing genes such as Crsp3 are upregulated in the border zones of ST 88. The downregulation of the Crsp3 gene exacerbates functional impairment after MI, while its upregulation improves this situation. Thus, CSRP3 may serve as a therapeutic target for myocardial infarction. Furthermore, ST also identified potential regulatory factors that influence myocardial cells and fibroblasts by comparing MI and normal cardiac tissue 92. Cardiac fibrosis plays a crucial role in the etiology of heart failure. In early studies, through Tomo-seq of ischemic hearts, SOX9 was identified as a potential therapeutic target for mitigating cardiac fibrosis 93. Recent ST analysis of mouse models revealed that the downregulation of Htra3 promotes cardiac fibrosis, and further integration with perturbation techniques and scRNA-seq analysis indicated that Htra3-TGF-β-IGFBP7 could be a therapeutic target for heart failure 94. Additionally, the molecular mechanisms governing the progressive loss of cardiomyocytes and their replacement by adipose cells in ACM have been elucidated through Tomo-seq analysis 90. This study identified ZBTB11 as a crucial factor responsible for inducing CM apoptosis. Considering this discovery, the inhibition of ZBTB11 has emerged as a promising therapeutic strategy for managing ACM.

In research on vascular diseases, ST has demonstrated utility in the identification of prospective targets associated with atherosclerosis and vasculitis 95,96. Arterial atherosclerotic plaque rupture precipitates acute cardiovascular complications. ST analysis of the rupture-prone regions within vascular plaques has elucidated the molecular mechanisms underlying arterial atherosclerotic plaque rupture 96. Moreover, novel therapeutic targets, including noteworthy candidates such as MMP9, have been identified based on spatial localization. In the field of Kawasaki disease vasculitis research, researchers have applied ST to analyse murine coronary arteritis tissue. Researchers have shown that focal infiltration of immune cells is associated with the activation of NLRP3 and the production of IL-1β and IL-18, which also regulate vascular smooth muscle phenotypic transition to the pathogenic type 95. This compelling evidence supports the potential of NLRP3 as a promising therapeutic target for vasculitis in Kawasaki disease patients.

### 3.4 Infectious diseases

The application of ST in infectious diseases aims to gain further insights into cellular interactions and retain spatial information vital for deciphering the interplay between different cell types post infection. Recent research on coronavirus disease 2019 (COVID-19) to understand the cellular causes associated with lung damage and identify therapeutic targets is a notable application of ST approaches in infectious diseases. By comparing gene expression data from 46 areas of lung samples affected by COVID-19 across 3 patients, ST has been applied to describe the atlas of damage in lung tissue affected by COVID-19, encompassing cells, pathways, and genes 97. Building upon this preceding study, the research team employed a combination of multi-omics analysis and machine learning techniques to delve deeper into their study. Through selective ST, they revealed signals associated with epithelial-mesenchymal transition and pinpointed LZTFL1 as a prospective therapeutic target 98. Another significant application of ST in infectious diseases is its use in studying influenza infection. ST has been employed in combination with scRNA-seq and bulk RNA-seq to reveal substantial changes in gene expression patterns among various immune, endothelial, and epithelial cell types, delineating differences in the host response to influenza infection between young and aged mice 99. The heightened levels of inflammation and fibrosis within the lungs of aged mice are clearly discernible through the perspective of ST. Furthermore, ST have highlighted the enrichment of Wfdc17 in fibrotic regions, which has been identified as a novel potential regulator following postinfluenza A virus infection. In the study of bacterial infections, ST has been used to map the spatial location of cell types within leprosy cell groups and the architecture of granulomeres 100. Moreover, a specific set of genes encoding antimicrobial proteins was identified; these genes are differentially expressed in disseminated leprosy and are controlled by the actions of IFN-γ and IL-1β. In another example of bacterial infection, the integration of spatial and temporal transcriptomics was employed to analyse a mouse model of endotoxemia 101. Through ST, the study revealed the spatial localization of the S3 proximal tubule subtype in the kidneys affected by endotoxemia. Additionally, temporal transcriptomic analysis revealed potential therapeutic targets for the treatment of human sepsis, including SOX9. ST also has applications in parasitic infectious diseases. Lymphatic filariasis is an ailment caused by nematodes, yet current drugs solely target the larvae, leaving the adults unaffected. Researchers have conducted ST analysis of the head region of adult parasites, establishing a gene expression map aimed at identifying potential novel drug and vaccine targets 102.

### 3.5 Other diseases

The kidney is a complex organ comprising a diverse array of structures with more than 20 distinct cell types in its mature state. The exploration of various intercellular communication networks and regional gene expression patterns within the kidney facilitates an enhanced understanding of renal diseases and the identification of potential therapeutic targets. Several studies employing ST in the field of kidney disease have undertaken comprehensive assessments of human and mouse model samples to identify potential therapeutic targets for kidney injury 103, chronic kidney disease 104, and kidney-related disorders 104-107. For example, in renal transplantation, T-cell-mediated rejection initiates an immune attack on the transplanted kidney, resulting in renal injury. The utilization of ST to interrogate the transplant region represents a promising strategy for identifying prospective therapeutic targets associated with acute cellular rejection or other localized renal diseases 108. In addition, renal fibrosis serves as a hallmark of the progressive progression of chronic kidney disease, yet the current therapeutic options for treating fibrosis are inadequate. The utilization of a strategy combining ST with scRNA-seq has elucidated the cellular origins and different roles of human renal myofibroblasts and their precursors 105. Additionally, this study identified NKD2 as a specific therapeutic target for renal fibrosis involving myofibroblasts. Recently, ST has also been applied in the discovery of intrinsic TGF-β signalling that reduces proximal tubular mitochondrial injury and inflammation in chronic kidney disease. 104 Acute kidney injury exhibits regional specificity. The application of ST visually elucidates the re-expression of region-specific markers lost during the process of repair 103. This method introduces a novel tool for identifying therapeutic targets in the context of acute kidney injury. In the field of diabetic kidney disease (DKD) research, researchers have employed ST and scRNA-seq to construct a renal atlas of DKD to unveil potential therapeutic targets for the treatment of DKD 107. In renal papillary samples from patients with kidney stones, ST analysis revealed diffuse expression of MMP7 in the stone area and upregulation of MMP9 in the mineral deposition region, suggesting that these two molecules may serve as novel biomarkers for kidney stones 106.

Hepatic function in drug metabolism is pivotal, and investigating hepatic processes has considerable implications for drug discovery and development. ST reveals the spatial distribution of cellular phenotypes and transcriptional profiles in hepatic disorders, thereby aiding in the understanding of pathogenic mechanisms 109. For example, analysing ST atlases of primary hepatocellular carcinoma can discern intratumoral diversities, encompassing predominant gene expression, functionalities, prognoses, and clonal origins of diverse cellular subpopulations, thereby facilitating the identification of therapeutic targets 110. Additionally, the ST shows the spatial arrangement of distinct cellular populations within the liver, thereby contributing to the exploration of hepatic tissue architecture and function. For instance, the transcriptional differences between hepatocytes and nonparenchymal cells have been discerned by ST, revealing their respective contributions to hepatic homeostasis and delineating gene expression profiles and structural constituents across the murine hepatic zone 111. Notably, in addition to scRNA-seq, ST has significant applications in the study of drug impacts on hepatic physiology, particularly drug-induced hepatotoxicity. For example, molecular distinctions between periportal and pericentral hepatocytes following exposure to acetaminophen have been elucidated by scRNA-seq, revealing the molecular underpinnings of acetaminophen-induced hepatic injury 112. ST analysis of hepatic tissue sections after drug administration enables researchers to discern alterations in gene expression within specific hepatic regions induced by distinct pharmaceutical agents. Correlation of regional tissue damage assessments with spatial expression aids in elucidating the toxic mechanisms of drugs and the pathophysiology of hepatic injury. A study integrating single-nucleus RNA sequencing and ST revealed a spatially dependent dose-response relationship between liver lipid accumulation and inflammatory responses induced by 2,3,7,8-tetrachlorodibenzo-p-dioxin (TCDD), elucidating the specificity of cellular phenotypes and transcriptional alterations in various hepatic regions induced by TCDD and the molecular mechanisms underlying TCDD-induced nonalcoholic fatty liver disease progression 113.

## 4. ST for drug research

**Figure 4:**
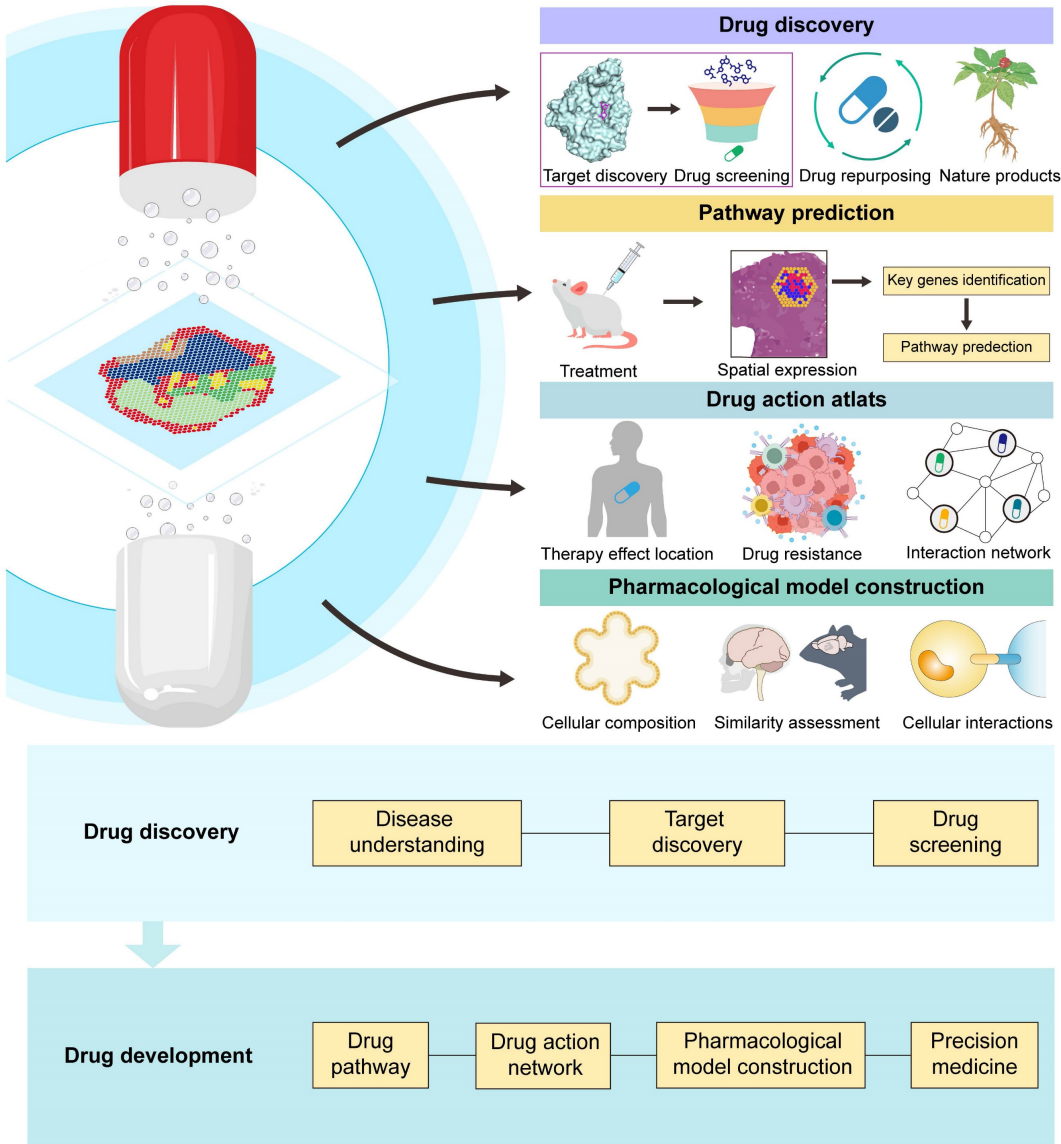
**Application of spatial transcriptomics in drug discovery and drug development.** ST aids in identifying new targets, facilitating drug screening. ST holds immense potential in drug repurposing and natural product drug discovery. ST utilizes spatial expression maps after drug treatment to identify key genes and predict treatment pathways. ST constructs a drug action atlas, enabling the localization of drug effects and the study of drug resistance and interactions. By analysing the spatial cell composition of pharmacological models, assessing model similarity to cases, and studying intercellular interactions, ST assists in building pharmacological research models. ST is being applied across various stages of drug discovery and development, and these applications are enhancing the probability of drug discovery success and improving therapeutic outcomes.

ST is driving incremental improvements in our pharmacological knowledge. This technology aids in identifying different cell types and regions associated with diseases, guiding the discovery of potential therapeutic drugs. Moreover, ST allows us to map changes in cells within specific areas and link them to their gene expression profiles. This helps identify genes and pathways involved in drug responses, offering a more precise understanding of how drugs work at the molecular level in distinct tissues or cell types. Although ST has not yet been widely applied in drug development, based on existing research, we can still foresee its enormous potential in future drug studies, particularly in pharmacology research. Hereafter, our discussion will focus on the application of this approach in drug discovery, pharmacological research, and precision medicine with several study cases, showing the utility of ST in drug development (Figure 4).

### 4.1 Drug discovery

Employing ST to pinpoint targets and then proceeding with drug identification and validation is a feasible approach for drug screening. Analysing unique gene expression patterns within affected regions and assessing cell interactions, along with leveraging established biological databases, enhances the precision of drug screening processes. For example, to study the prognosis and therapeutic strategies for cutaneous melanoma, researchers conducted an analysis of publicly available human cutaneous melanoma ST data from the 10x Genomics database, aiming to identify specific cellular types and gene expression patterns within distinct regions of melanoma 114. Subsequently, the team employed single-cell transcriptomic analysis to validate characteristic genes associated with cancer-associated fibroblasts. Following this validation, they screened small molecular compounds from the CTD database capable of downregulating the gene expression of FBLN1 and COL5A1. Using the AutoDock Vina molecular docking method, they screened and performed molecular docking to identify potential drugs targeting FBLN1 and COL5A1, such as mifepristone and dexamethasone. Applying ST to discover new pathways influencing diseases can help expand the therapeutic scope of known drugs into novel areas of disease treatment, thereby granting these existing medications new application value. In a study with a mouse gastric tumor organoid model, researchers identified a set of key genes expressed in stromal cells located at the invasive front of tumors through ST analysis and discovered that MAPK signal transduction promotes tumor metastasis 115. Based on these findings, researchers have attempted to use the MAPK kinase inhibitor trametinib to inhibit the development and invasion of gastric tumors. ST has also been applied in the discovery of natural active compounds. There are currently no effective pharmacological treatments for hepatic ischaemia-reperfusion injury (I/R) 116. Researchers have conducted an analysis integrating ST with histopathological methods to scrutinize molecular and cellular changes within the injured hepatic area. Subsequently, leveraging region-specific gene expression variations, they queried the Connectivity MAP database for genes demonstrating I/R modulation and regional dependency characteristics, which led to the identification of the natural active compound celastrol as a potential therapeutic agent for I/R 117. Further validation of celastrol's regulatory effects within relevant ROIs was also conducted using ST in this study. ST has been employed to identify potential drugs associated with breast cancer tumor tissue. Elevated levels of inner mitochondrial membrane protein (IMMT) were found to be coexpressed within the transformed sections of breast cancer tumor tissue by ST 118. By leveraging the expression of IMMT, researchers have screened the Genomics of Drug Sensitivity in Cancer (GDSC) and Cancer Cell Line Encyclopedia (CCLE) databases, and pyridinol was identified as a potential drug candidate.

Advances in computer science and artificial intelligence have propelled the application of ST in drug screening. In the research of oral squamous cell carcinoma (OSCC), a study used the ST method to explore the gene expression variances within the tumor core (TC) and leading edge (LE) regions of OSCC and further identified potentially effective drugs that could impede the transfer from TC to LE 119. Based on RNA velocity, Scvelo, a dynamic simulation model, was used to analyse cellular developmental trajectories and gene expression dynamics 120. Dynamo is a machine learning technique used to predict cellular fate transitions following genetic perturbations 121. By integrating both approaches, researchers characterized the developmental trajectories of cancer cells in the tumor core (TC) and edge (LE) regions using Dynamo to associate them with drug responses via the integration of gene-specific transcriptional dynamics. A total of 417 drugs were analysed, revealing 140 drugs demonstrating drug-gene interactions. This study has presented a novel approach to the application of ST in drug screening by integrating ST with the in silico technique. In another study, ST was used to examine the correlation between kidney injury and kidney renal clear cell carcinoma (KIRC), with a focus on alterations in the expression of the PGAM1 gene in both kidney injury and KIRC 122. Q-omics, an integrative omics data mining software comprising data mining and visualization algorithms, was applied 123. In this study, Q-omics was applied to screen the Genomics of Drug Sensitivity in Cancer (GDSC) database, and Pearson correlation coefficient analyses between PGAM1 expression levels and drug dosages were conducted to assess the sensitivity of various drugs in the context of high PGAM1 expression, identifying four drugs with potential therapeutic efficacy.

### 4.2 Pharmacological research

#### 4.2.1 Drug pathway prediction and validation

The ST not only accelerated the discovery of new drugs by providing precision and spatial information but also aided in understanding drug mechanisms by predicting drug action pathways. Biological pathways and interactions within organisms are remarkably diverse and intricate, especially regarding drug actions involving various molecular interactions. Moreover, some drug mechanisms might involve biological processes that are not fully understood yet, further complicating pathway prediction. ST provides specific location information regarding drug actions within tissues, encompassing varied responses among different cell types and regions. This spatial resolution of cellular and tissue interaction information contributes to overcoming barriers in deciphering complex drug action mechanisms. The precise mechanism underlying the prolonged antidepressant effects of ketamine through facilitating myelin sheath formation remains elusive based on earlier research. With the aid of ST, researchers have investigated the enduring impact of ketamine treatment on the medial prefrontal cortex and hippocampus in mice, identified crucial DEGs involved in myelin sheath formation and revealed the pivotal role of the AMPAR pathway in mediating myelination 124. This unveiling through ST shed light on the sustained antidepressant mechanism of ketamine. Similarly, in the field of vaccine therapeutics, ST revealed the CD4-dependent expression of interferon-stimulated genes in lung myeloid cells and epithelial cells and revealed that the Bacillus Calmette-Guérin vaccine induces CD4+ T cells to provide feedback to tissue myeloid cells and epithelial cells to achieve sustained broad-spectrum immune mechanisms 125.

Numerous traditional natural remedies have demonstrated significant therapeutic efficacy, yet their precise molecular mechanisms remain unclear. The application of ST has substantial potential for predicting the pathways of action of active constituents in natural remedies. For example, an ST analysis of a mouse brain revealed the molecular pathways influenced by Rhynchophylline, an alkaloid component of Uncaria known for its significant sleep-inducing effects, indicating that Rhynchophylline impacts genes associated with sleep regulation in specific brain regions 126. Compared to single-component drugs, traditional Chinese medicine (TCM) is characterized by multiple targets, multiple pathways, and holistic regulation, thereby resulting in greater complexity. Notably, ST can capture the interactions of TCM between cells and tissues, assisting in revealing the intricate mechanisms of TCM action and the patterns of multipathway regulation, making it a powerful tool for revealing new pathways of TCM. To elucidate the mechanism by which the traditional Chinese medicine formula Shexiang Baoxin Pill (SBS) protects the heart, researchers have combined ST with single-nucleus RNA sequencing to assess the effects of SBS on myocardial cell types and subpopulation states 127. They discovered that SBS could increase the gene expression of Nppb and Npr3 in the infarct area of the heart, regulating vascular generation mediated by endocardial cells. However, this study did not further validate this pathway. Despite the enormous potential of ST in traditional Chinese medicine, its current application in TCM research remains limited.

Additionally, in terms of validating drug pathways, ST has the ability to capture characteristic spatial differences following drug action. Through the combined application of single-cell transcriptomics and ST, it was observed that the interaction between tumor cells and immune cells (particularly the increased interaction between CD8+ T cells and tumor cells) was strengthened after NP137 treatment, further confirming the inhibitory effect of NP137 treatment on Netrin-1 and subsequently reducing EMT in tumors 128.

#### 4.2.2 Panoramic and dynamic depiction of the drug action network

ST offers an unprecedented opportunity to gain a more detailed view of drug action regions at the molecular level within tissues or cells, enabling the construction of dynamic panoramic drug action networks and offering novel insights into drug efficacy assessment. In a previous study of prostatic hyperplasia, a large population of differentiating cells was identified to facilitate prostate regeneration using scRNA-seq 129. Nevertheless, this study did not explore the impact of intercellular communication and the microenvironment on the status of prostate cells. With the application of ST in heterogeneous response studies to drug treatments, the molecular characteristics associated with the heterogeneous response to 5-alpha reductase inhibitor (5ARI) therapy for benign prostatic hyperplasia have been identified 130. This study revealed a transition from prostate epithelial cells to club-like cells following 5ARI therapy and revealed that this transition was strongly associated with the degree of glandular atrophy. ST also provides a promising tool for identifying spatially relevant regions of drug action within the tumor microenvironment that have biological and therapeutic implications 131. In the first study that mapped spatially resolved gene expression in Sonic hedgehog (SHH) patient-derived orthotopic xenograft (PDOX) medulloblastoma, a reduction in cellular diversity within the tumor following treatment with the CDK4/6 inhibitor palbociclib was revealed 132. Interestingly, despite palbociclib treatment, cells at the tumor-microenvironment interface continue to proliferate, suggesting that cells specific to transcriptional states or spatial positions play a critical role in the response to palbociclib therapy. This finding elucidates the reason behind the recurrence observed in 80% of SHH PDOX tumors following cessation of palbociclib treatment in earlier studies 133. A clear example of the use of ST analysis in assessing the potential effects of natural active compounds is the assessment of the potential effects of 47 ROIs in I/R-induced liver injury, which revealed that celastrol mitigated the infiltration of macrophages induced by I/R in specific injury zones and partially restored the characteristics of certain hepatic nonparenchymal cells affected by I/R 117. Accurate drug targeting enhances the efficacy of medications. Through the integration of snRNA-seq and ST, researchers revealed the precise localization of prostaglandin E2 receptors, revealing that the site at which prostaglandin E2 is injected during uterine fibroid resection surgery should be at the junction of pseudocysts and smooth muscle tumors, leading to a greater reduction in bleeding 134.

ST has a unique advantage in deciphering intricate interaction networks between drugs in combination therapies and between drugs and the organism. Combination therapy has been effectively employed in cancer treatment to reduce drug resistance and enhance therapeutic efficacy for patients. However, numerous unanswered questions persist regarding the mechanisms underlying the improved drug efficacy achieved through combination therapy. In the field of combination therapy with immune checkpoint inhibitors (ICIs), identifying the tumor immune interactions that contribute to the response to ICIs remains a pivotal objective. A recent study examined the spatial transcriptomic profiles of T cells, stromal cells, and leukemia cells within the treatment sites of acute myeloid leukemia (R-AML) patients receiving pembrolizumab and decitabine combination ICI therapy and elucidated the impact of pembrolizumab and decitabine combination therapy on the tumor immune microenvironment 135. Identifying new predictive biomarkers for tumor resistance in specific regions will aid in the exploration of new combination therapy approaches to enhance therapeutic efficacy. During the investigation of intraductal papillary mucinous neoplasms (IPMN), an analysis revealed an increase in mucin O-glycosylation pathways throughout IPMN progression and revealed that tantalate can target GCNT3 for mucin regulation and that combining tantalates with chemotherapy enhances immune infiltration, leading to improved therapeutic efficacy 136. Due to its capacity to construct more comprehensive and accurate dynamic maps, there is increasing interest in the application of spatiotemporal transcriptomics in drug action research. However, their current application in pharmacological research remains relatively limited.

#### 4.2.3 Pharmacological model construction

Organoids are three-dimensional cell clusters constructed with multiple cell types through the induction and self-assembly of human tissues, stem cells, or pluripotent stem cells in vitro 137. Within each individual organoid, diverse cell types resembling natural human organs are present. The remarkable similarity in both function and structure to those of native human organs makes organoids a robust platform for studying human disease mechanisms and drug screening 138. Cell culture systems are mainly classified into 2D and 3D culture systems 139. Compared with traditional 2D cell models, organoids exhibit exceptional biological similarity 140. Additionally, compared to animal models, organoids offer reduced culture expenses and significant advantages in high-throughput screening 141. Furthermore, they can faithfully recapitulate human organ development characteristics and do not exhibit species differences 142,143. Consequently, organoids have found extensive applications in diverse fields, including disease modelling 144, drug screening 145, drug evaluation 146, toxicity assessment 147, and cancer research 148. In September 2022, the U.S. Food and Drug Administration (FDA) issued new guidelines, urging the reduction of animal testing and the adoption of novel technologies as alternatives whenever possible, with organoids having emerged as a high-quality substitute for preclinical animal experiments 138,149. To determine the heterogeneity of organoids, single-cell sequencing has been employed in organoid research. Single-cell sequencing allows researchers to determine transcriptional characteristics and identify cell types within organoids that closely resemble those found in actual organs 150-152. Nevertheless, these methods lack the ability to provide precise spatial transcriptional information 153. ST, in contrast, offers the ability to decipher the spatial distribution and transcriptional characteristics of various cell types within organoids 154. Currently, ST primarily provides assistance in three key aspects within the realm of organoids: 1). Understanding the spatial composition of cell types within organoids; 2). The spatial expression profiles of the organoid transcriptome were explored; 3). Investigating the intercellular interactions within organoids. However, the application of spatial transcriptomes in organoid research remains unpopular.

The formation of organoids closely mimics the characteristics and cellular composition of real organs, thus recapitulating the self-organizing processes of organ development. Therefore, studying human organ development through organoids in vitro offers a higher level of fidelity compared to that of model organisms such as mice or cell-based models 155. From another perspective, gaining an understanding of organ development patterns in human embryos is also advantageous for the cultivation of organoids 156. The application of scRNA-seq in developmental biology enables the comprehensive analysis of tissue transcriptomes at single-cell resolution across various stages of development 157,158. However, current organoid analysis approaches still have certain limitations, and addressing how to depict the organoid development process more faithfully remains a challenge. For example, due to the nonfixed composition of organoids and the heterogeneity both between and within organoids, the need for integrating multiple technologies for high-throughput analysis in dynamic organ development models remains unmet 159. In the study of neurological disorders, the human brain possesses unique characteristics distinct from those of the brains of other animals 160. Cerebral organoids emulate the tissue architecture and multilineage differentiation seen in the human brain, offering a new window into investigating human brain development 161. Single-cell transcriptomic analysis throughout the entire human brain organoid development process, from the pluripotency to neuroectoderm and neural epithelial stages, enables the understanding of the cellular composition of organoids and the reconstruction of differentiation trajectories 162. Similarly, comparing the transcriptomes of human cortical organoids and fetal brains can validate the authenticity of organoid models 163. However, previous single-cell transcriptomics approaches have not been able to capture the dynamic spatial changes in the transcriptome during developmental processes. In lineage tracing of human cerebral organoids, cellular populations in different regions of the brain may be associated with clonal spatial arrangements, while ST serve as a bridge connecting molecular states, cellular lineages, and spatial positional information 164. Spatial transcriptomics has also been widely used in retinal organoid development. Retinal organoids are complex organoids containing a rich population of rod and cone cells, and they are capable of recapitulating the human retina's developmental process from the optic vesicle to the optic cup 165,166. The development of retinal organoids is strongly correlated with retinal differentiation programs and genes related to development, both in terms of their chemical composition and cellular makeup 167. The integration of ST with single-cell transcriptomics and temporal transcriptomics has enabled the precise reconstruction of the process of retinal neuron differentiation and localization during retinal organoid development 168. Through multiple smFISH methods, researchers obtained spatial transcriptomic information and inferred the gene regulatory networks underlying retinal organoid development 169.

In addition, the study of organ development to achieve higher-fidelity construction of organoid models remains a research direction worth exploring. Legnini et al. devised a method for spatiotemporally programming organoids to establish unique gene expression domains, validated this approach using ST, and revealed the role of the SHH pathway in an organoid model of human neurodevelopment 170. In the study of kidney development, spatial and temporal transcriptomic analysis of human embryonic kidneys has been employed to elucidate intercellular signalling during organoid development and the key factors that regulate kidney development, thus facilitating the cultivation of kidney organoids 156.

### 4.3 Precision medicine

Due to variations in genetics, physiology, biochemistry, and other factors among different individuals, patients exhibit diverse responses to the same disease and the same drug treatment 171,172. Some individuals might be highly sensitive to a particular medication, while others may show insensitivity or even severe side effects to the same drug 173. Therefore, there is a need to determine a better treatment approach based on individual characteristics to enhance treatment effectiveness and reduce adverse reactions. ST has emerged as a powerful tool for precision medicine, facilitating precise diagnosis and targeted therapies 174. The precise spatial localization capability of ST enables accurate examination of biomarker variations subsequent to drug administration across various patients, aiding in patient stratification for predicting individual responses to treatment. In a study of the tumor immune microenvironment, scRNA-seq and ST datasets from various patients and tumor types were analysed, leading to the establishment of a tumor immune atlas for precision therapy 175. The comprehensive single-cell atlas of the spatial location of gastric cancer delineates high-resolution maps between patients with various subtypes of the same gastric cancer and across different subtypes, identifying key factors predicting adverse reactions related to prognosis 176. The combined therapy of antiangiogenic therapy and immune checkpoint inhibition is effective against HCC but lacks universal efficacy across all patients, with no identified biomarkers to differentiate responders from non-responders 177,178. An ST-analysis of tumor samples from patients with HCC treated with cabozantinib and nivolumab revealed significant crosstalk between cancer cells and the tumor microenvironment, which plays a pivotal role in tumor response and drug resistance, and identified biomarkers capable of predicting patient responses, offering new avenues for precise classification and therapeutic strategies 179. With a combination of scRNA-seq, RNA-Mutect, and ST, circulating lncRNAs associated with chemotherapy resistance in plasma have been identified as novel predictive circulating biomarkers for the progression of lung adenocarcinoma 180. To predict the chemotherapy response in advanced hepatobiliary cancer patients and elucidate the molecular mechanisms underlying drug resistance, researchers have analysed the transcriptomic characteristics of rapid progressors (RP) and long-term survivors (LS), proposing the RPLS metric as a novel indicator of the effect of chemotherapy 181.

**Figure 5:**
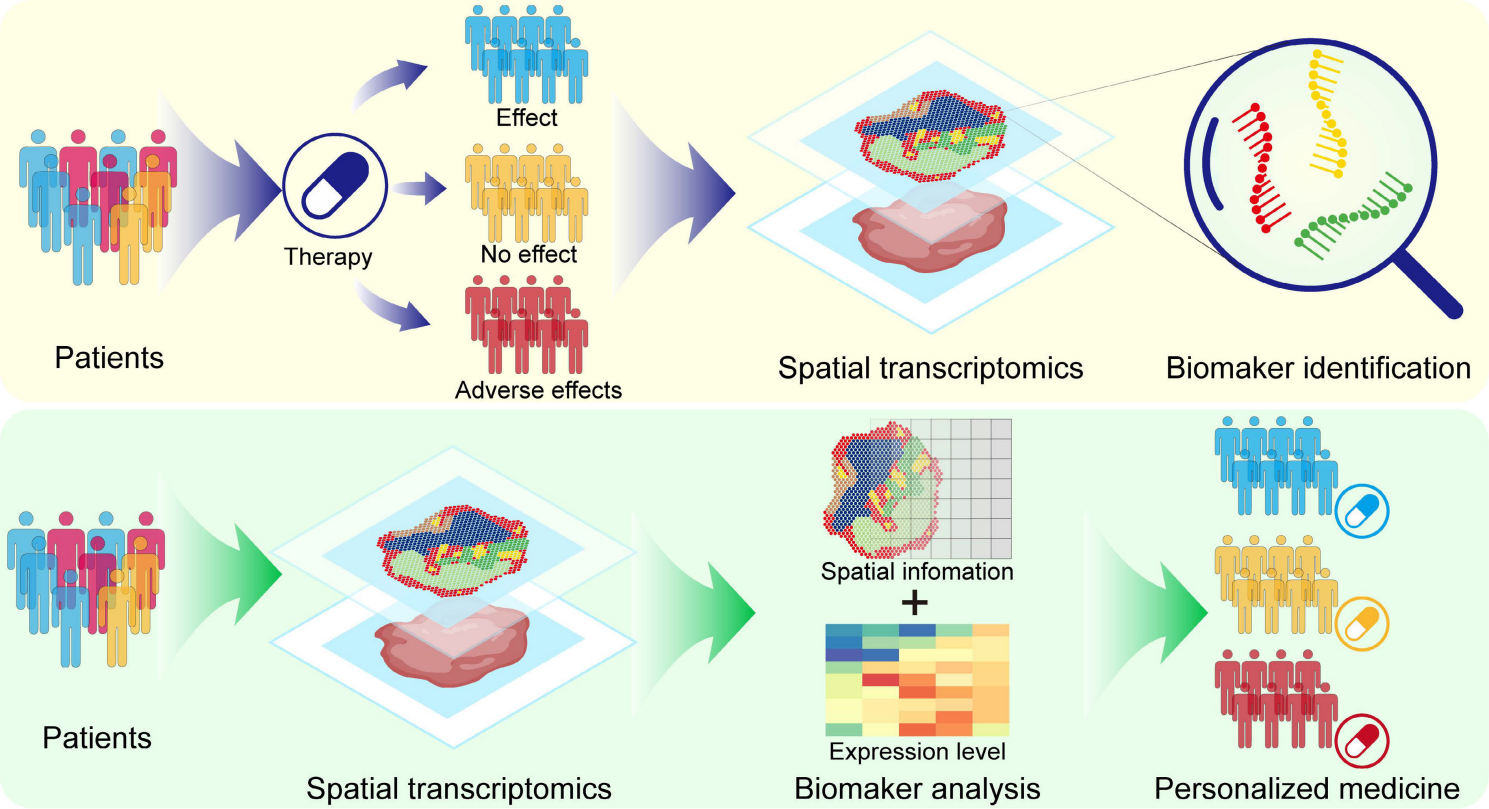
** Spatial transcriptomics in personalized medicine.** ST identifies biomarkers of drug response by analysing the spatial transcriptome information of different drug-responsive populations. The spatial transcriptome was used to analyse the spatial expression information of patient biomarkers to achieve personalized medicine.

ST can precisely identify and locate therapeutic targets suitable for individual patients and validate the effectiveness of these targets. A close relationship exists between senescent cells and malignant tumors 182. Senescent cells, on the one hand, express age-related genes that inhibit tumors, while on the other hand, age-related genes facilitate tumor growth, invasion, and metastasis 183. In a recent study, three distinct tumor aging microenvironment (AME) regulatory patterns and 36 AME regulatory factors were identified through ST analysis of the AME in prostate cancer, providing potential targets for personalized therapies 184. In patient tissue biopsies, ST has the potential to provide precise insights into the molecular mechanisms underlying disease progression. A study in precision medicine related to the kidneys analysed the gene expression characteristics of a biopsy tissue region from a diabetic patient, discovering the molecular mechanisms of neovascularization formation in the glomeruli, thereby introducing novel personalized diagnostic methods for renal biopsies 178. ST analysis of biopsy material from primary central nervous system lymphomas revealed the spatial heterogeneity of malignant B-cell clusters and revealed the colocalization of T-cell exhaustion markers with malignant cells, demonstrating the potential of ST in personalized therapy 185. In summary, analysing ST information aids in the development of personalized drugs or treatment strategies tailored to individual pathological features (Figure 5).

## 5. Challenges and prospects

### 5.1 Technological limitations and advancements

At the technological level, current spatial transcriptomic techniques are still in their early stages. Despite ongoing technological advancements, there are still limitations in spatial resolution compared to single-cell transcriptomic techniques 186. ST still cannot provide sufficiently precise information for certain subcellular structures within cells or areas with small intercellular distances. To address this issue, researchers have developed a series of approaches for integrating single-cell transcriptomic and spatial transcriptomic data 187. Additionally, spatial transcriptomic resolution can be enhanced through algorithms such as the BayesSpace algorithm, which is based on a fully Bayesian statistical approach 188. This algorithm utilizes spatial neighborhood information to improve the resolution of spatial transcriptomic data to the level and models the low-dimensional representation of gene expression to achieve spatial clustering, significantly enhancing the utilization of spatial information. Resolution can also be improved by optimizing the technology for capturing transcriptomic information. The Visium HD platform recently introduced by 10X Genomics, for example, has reduced the diameter of spots from 55 micrometers to an edge length of 2 micrometers and eliminated gaps between spots. This significant enhancement enables gap-free and bias-free ST at single-cell resolution.

Furthermore, current mainstream spatial transcriptomic techniques are mostly based on two-dimensional tissue sections, which can obtain two-dimensional spatial transcriptomic information but still lack adequate capability for acquiring transcriptional information in three-dimensional space. An R language toolkit called STUtility has been developed, which can automatically perform data conversion, align multiple tissue slices, and regionally annotate continuous two-dimensional slice data, ultimately creating a final three-dimensional model of tissues from processed sequencing and image data 189. However, this method still relies on computer algorithms for three-dimensional model construction and still faces challenges related to ensuring slice quality and discrepancies between constructed three-dimensional models and actual scenarios. Addressing this issue requires revolutionary technology to develop methods that can directly capture three-dimensional spatial transcriptomic information. Imaging-based spatial transcriptomic methods can directly observe three-dimensional tissues and theoretically hold more potential and advantages in three-dimensional ST. For example, as mentioned earlier, STARmap analyses three-dimensional transcriptomic data using hydrogel and tissue-clearing technologies 46. The key to this approach lies in enhancing imaging techniques to observe more comprehensive positional signals and decoding them to construct comprehensive three-dimensional transcriptomic maps.

The current mainstream platform for ST is the commercialized 10X Genomic Visium Spatial Gene Expression platform. However, due to the high cost, operational complexity, and requirement for specialized equipment, its widespread application and promotion in drug research and personalized therapy are restricted. Encouragingly, ST, as a trending technology in recent years, has continued to evolve with the emergence of new methodologies 190. It is believed that in the foreseeable future, there will be more affordable and user-friendly solutions to lower the threshold of this technology.

### 5.2 Integration of ST with AI

With the rapid advancement of ST, a substantial volume of spatial transcriptomic data has accumulated. However, the current challenge lies in improving the efficiency of data acquisition and extracting valuable knowledge from this extensive dataset. Consequently, there is an urgent need for data standardization and the establishment of databases to facilitate the sharing of spatial transcriptomic data and streamline the data acquisition process for researchers. Additionally, the application of artificial intelligence (AI), such as machine learning (ML) and deep learning (DL) methodologies, can assist in uncovering potential drug targets and drug effects from the vast spatial transcriptomic dataset, maximizing the exploration of valuable spatial transcriptomic data resources. With respect to database establishment, researchers have already developed several comprehensive spatial transcriptomic databases 191-194, and future databases will integrate more analytical tools to achieve a one-stop solution for data collection and analysis.

AI technologies contribute to the computational analysis of spatial transcriptomic data, offering limited possibilities for the advancement of ST. DeepST is a DL-based ensemble algorithm that can accurately identify regions with similarities in gene expression and tissue morphology 195. The utilization of AI for the analysis of ST data contributes to enhanced resolution. By way of illustration, the SCS method employs a transformer architecture to more accurately and efficiently segment individual cells from high-resolution ST images and even enables the analysis of heterogeneity within individual cells 196. A recent study introduced SpatialScope, a method employing deep generative models to integrate single-cell transcriptomic and spatial transcriptomic data 197. This approach not only enhances the single-cell resolution of sequence-based spatial transcriptomic data but also infers complete transcriptomic expression levels based on image-based spatial transcriptomic data. Artificial intelligence ingeniously provides a novel approach to address the challenges in constructing three-dimensional transcriptomic maps traditionally associated with ST. STitch3D innovatively combines probability models with deep neural networks to simultaneously analyse multiple consecutive spatial transcriptomic slices, thereby reconstructing three-dimensional tissue structures 198. A series of AI tools have been developed, including STAGATE for identifying spatial substructures in biological tissues 199, STAligner for integrating and analysing multislice spatial transcriptomic data 195, and STAMarker for spatial domain recognition and identification of spatially variable genes 200. This series of tools integrates artificial intelligence models to improve the efficiency and accuracy of computing and analysing spatial transcriptomic data.

### 5.3 Spatial multi-omics and spatiotemporal transcriptomics

Integrating ST with spatial metabolomics, spatial proteomics, and similar technologies facilitates the simultaneous acquisition of spatial distribution information for gene expression, metabolites, and proteins 201,202. The combined analysis of single-cell transcriptomics, ST, and spatial proteomics has been employed to elucidate multicellular mechanisms underlying the progressive onset of Alzheimer's disease and their spatial relationships with stages of neurodegenerative changes 203. The integration of ST with spatial metabolomics has enabled the construction of comprehensive spatial expression maps for genes and metabolites in complex human brains affected by injury 204.

Moreover, spatial-temporal transcriptomics technology enables the acquisition of dynamic and comprehensive information but has been extensively applied in developmental biology, but its potential in drug research remains largely unexplored 205-207.

### 5.4 Potential application of ST

Although ST has been applied in discovering disease targets, predicting drug action pathways, and delineating spatial maps, its potential in the field of drug development extends far beyond these aspects. Furthermore, despite the limited application of ST in natural medicine, its ability to construct comprehensive post-drug transcriptomic change maps holds promise as a robust tool for evaluating the effects of complex medications, predicting drug interactions, and potential pathway assessments. Hence, the field of natural medicine research, particularly in traditional Chinese medicine pharmacology, stands as a highly promising area for the application of ST. Pharmacological models are advancing toward building models that closely mimic the human body. ST provides a spatial viewpoint for comprehending the cellular composition and molecular features of human organs, facilitating the advancement of more precise preclinical models. However, due to limitations in resolution and sample processing techniques, the current application of ST in organoids remains relatively limited.

**Figure 6:**
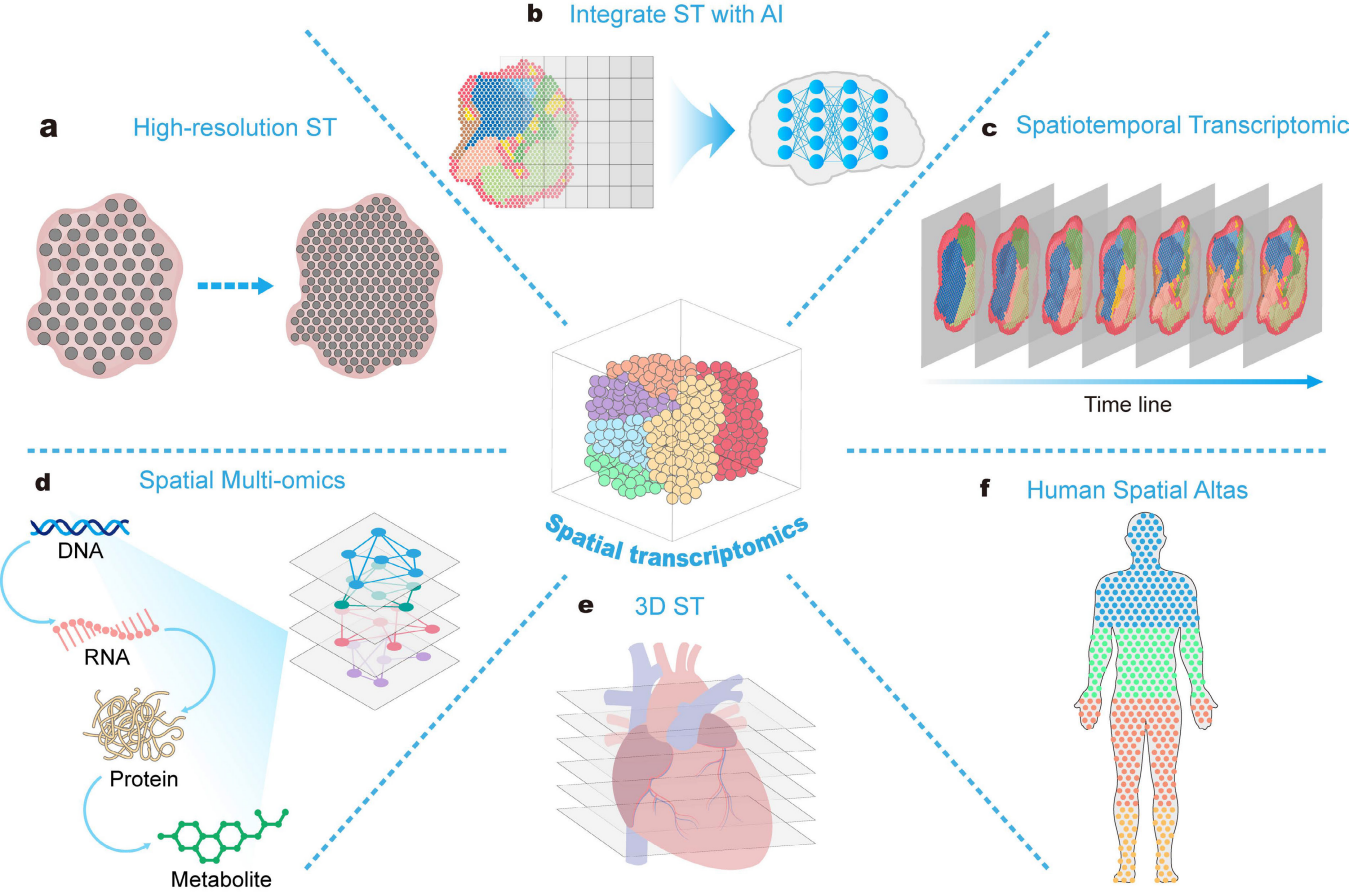
** Rapidly advancing spatial transcriptomics technology and its application prospects.** The integration of spatial transcriptome technology with other emerging technologies promotes understanding of disease and the construction of human spatial maps.

The ST is burgeoning and holds vast potential opportunities in the field of drug development, despite facing challenges such as data processing and technical limitations. In the future, advancements such as high-resolution ST, the integration of AI technologies with ST, three-dimensional ST, spatiotemporal transcriptomics, spatial multi-omics technologies, and human spatial mapping will greatly expand the application prospects of ST (Figure 6). These developments will offer more comprehensive insights for drug development, hastening the drug discovery process, and facilitating personalized therapy.

## 6. Conclusion

Overall, ST, as an emerging omics technology, holds immense potential in the field of drug discovery and development. With higher spatial resolution, ST effectively reveals gene expression at specific locations within tissue structures and their interaction relationships. This information is crucial for enhancing our understanding of diseases and discovering new therapeutic targets and drugs. Additionally, ST aids in establishing improved pharmacological research models, such as organoid models. Moreover, analysing drug pathways from a spatial resolution perspective can aid in more accurate prediction and validation of drug effects. The construction of dynamic panoramic networks of drug action further deepens our understanding of drug effects, particularly in combined therapies. Analysing the interaction of drugs within critical areas of a patient's body contributes to advancing personalized treatments, accelerating the discovery of new biomarkers, and providing more efficient treatment plans for patients. The distinct advantages in unraveling spatial information within complex tissues offered by ST, thereby providing crucial support for researching the distribution and mechanisms of action of complex drugs like traditional Chinese medicine. However, the current application of ST in drug development remains limited and faces certain challenges. Encouragingly, with the increasing maturity of high-throughput sequencing technologies, new opportunities are emerging, and ST may significantly alter the landscape of drug development.

## Figures and Tables

**Table 1:** Comparison of different spatial transcriptomics methods.

Method	Year	Resolution	Probes	Sample	Advantage	Limitation
**In situ capture**						
ST	2016	100 µm	Oligo probes	Fresh-frozen tissue	High throughput	Lower resolution
APEX-seq	2019	Subcellular (<10 µm)	Enzymatic tagging	Mammalian cells	Captures in situ activity; High temporal resolution	Requires specific enzyme for taggingLimited to cells with enzymatic activity
DSP	2019	10 µm	DNA Oligo probes	FFPEFrozen tissue	Profile both RNA and proteinsFlexibility in ROI selection	Limited to predefined panelsSpecialized equipment needed
HDST	2019	10-100 µm	Barcoded beads	Fresh-frozen tissue	High resolution Analyze large tissue areas	
Slide-seq	2019	100-200 µm	Unique molecular identifiers (UMIs)	Fresh-frozen tissue	High resolutionHigh sensitivity	
DBIT-SEQ	2020	Subcellular (<10 µm)	Barcoded probes	Fresh-frozen tissue	High resolutionCaptures both mRNA and protein	
Seq-Scope	2021	Subcellular (<10 µm)	RNA probes	Fresh-frozen tissue	High resolutionHigh compatibility	
Slide-seqV2	2021	10-20 µm	Barcoded probes	Fresh-frozen tissue	Improved resolution over Slide-seq;Can detect low-abundance transcripts	
Stereo-seq	2022	Subcellular (<10 µm)	Expansion microscopy	Fresh-frozen tissue	3D imaging capability	
Visium HD	2024	55 µm	Bead-based	FFPE, frozen tissue	High throughputAnalyze large tissue areas	
						
**Imaging-Based approaches**						
ISH						
FISH	1969	10-20 nm	DNA or RNA probes	Fixed cells or tissues	High specificity for DNA or RNA targets	Small number of targetsCross-hybridization and autofluorescence
smFISH	2011	Single-molecule	Oligo-dT probes	Fixed or live cells	Visualization of individual mRNA moleculesQuantitative analysis	Limited by the number of fluorophoresComplex imaging required
seqFISH	2014	Single-cell	Multiplexed probes	Fixed cells	Enables the detection of thousands of RNA molecules in a highly multiplexed manner	Complex image analysisHigher background signal
MERFISH	2015	Single-cell	Error-robust barcodes	Fixed cells	High multiplexing capabilityError correction	Requires high-quality imaging equipment and expertise
osmFISH	2018	Single-molecule	Multiplexed probes	Fixed cells	High multiplexingHigh sensitivity	Low signal-to-noise ratioRequire optimization for different samples
EEL FISH	2022	Single-cell	High-density probes	Fixed cells	High spatial resolutionLarge-scale gene expression analysis	
ISS						
FISSEQ	2014	Subcellular (<10 µm)	Fluorescently labeled probes	Fixed cells	Captures all types of RNA in situSubcellular levels	Lower throughputSmall field of view
BaristaSeq	2018	Single-cell	Padlock probes	Fresh-frozen tissue	High amplification efficiencySequencing accuracy of at least 97%	Requires specific equipment and expertise
STARmap	2018	Subcellular (200-300 nm)	Optimized probes	Fresh-frozen tissue	High-resolution 3D intact-tissue sequencing	Complex sample preparationNot suitable for all types of samples
BARseq	2019	Single neuron level	Viral encoding RNA barcodes	Frozen tissue, fixed tissue	High throughputNeuronal markers	Only for neuron
MERSCOPE	2022	100nm	Fluorescently labeled probes	FFPEFrozen tissue	High throughputNo sequencing required	
Xenium	2022	Subcellular (<10 µm)	Padlock probes	Fresh-frozen tissue	High sensitivity and specificitySupports customized gene panels	
						
**Other approaches**						
LCM	1996	Cell-level	None	Frozen or FFPE tissue sections	High precision in isolating specific cell types	Lower throughputLimited by size and structure of the tissue
TSCS	2016	Single-cell	None	FFPE	Assesses genomic copy number variations	Lower sequencing depth
TIVA	2014	Single-cell	None	Live tissue	ST analysis in vivoCaptures dynamic gene expression	Limited by the number of photoactivatable tags
RAINBOW-seq	2015	~1 micrometer	DNA probes	Fresh-frozen tissue	Enables multi-color imaging and quantitative analysis of mRNA	Complex probe design
